# Domestic Summary

**Published:** 1839-07-20

**Authors:** 


### DOMESTIC SUMMARY.

*Albany Medical College.*—From the circular and catalogue of this institution, we learn that a class of sixty-eight attended the late course, thirteen of whom received the degree of Doctor of Medicine.

*A case of Aneurism, successfully treated by the Needle. Read before the Medical Society of Georgia.* By C. P. Richardsone, of Savannah, Geo.—A. B., an Irish labourer, called at my office on the 27th of January last, with a tumour, the size of a pigeon's egg, two inches above the wrist; the arm was much swollen and tense; its temperature greatly elevated, and, as may be supposed, he was labouring under great constitutional disturbance. The tumour was aneurismal; produced by a wound of the radial artery, an inch below the aneurism. The history which he gave me of the case was, that in some fight, three weeks before, he had been stabbed. At the time, the wound bled considerably, but was stopped without surgical aid, and as the wound readily healed, no more was thought about it. In about a week afterwards, his arm became swollen from the elbow to the wrist, and very painful. The tumour then became obvious at the place I have mentioned. Its gradual increase, with the pain and tumefaction of his arm, induced him, two weeks afterwards, to seek advice. When I first saw him, his arm was much swollen with the blood that had infiltered throughout its whole cellular tissue; the heat and redness intense; his whole system participating in the irritation; and the aneurism tense and pulsating. It was at thi time that I passed the needle horizontally through the tumour—using the precaution to arm the extremities of the needle with pieces of cork, that it might not escape or be caught by the roller with which I encircled the arm. Directions were then given to keep the arm constantly flexed—the bandage to be saturated with cold water—to take a large dose of salts, and to refrain from food. Previous to the passing of the needle, the pulsation of the tumour and the radial artery below the tumour, was distinctly felt. But shortly afterwards (ten minutes) the diastole of the artery was barely perceptible, whilst the pulsating of the tumour had altogether ceased. On the 30th, three days after the first dressing, the tumour was hard, and no impression could be made by pressure—the pulsation of the artery below the tumour was very plain—the pain, heat and tumefaction of the arm had subsided, with no appearance of suppuration at the point at which the needle was introduced.

*2d of March.* The tumour was still hard, and there was no pulsation of the radial artery; round the place of entrance and exit of the needle were the appearances of suppuration. In one week from the time I passed the needle, the coagula had sloughed out, and the artery above the tumour, as far as could be ascertained, obliterated. Two weeks perfected the cure, and the man was able to go to work. Had the needle been withdrawn three days after its introduction, or before any signs of suppuration had made their appearance, I believe that the coagula would not have sloughed, but would have been absorbed, and the parietes of the sac gradually coalesced. I further believe, that whenever coagulation of the blood in an aneurismal tumour does take place, that the cure of the aneurism may be considered as accomplished, for, as I before said, the sac will contract, the coagulum will be absorbed, some portions in continuity with the sac will become engorged, and consolidate, others will escape by the process of ulceration through the integuments, and ultimately a progressive coalescence of the tumour will thus take place.

There were circumstances in the case just detailed that were far from being favourable to a fair trial of the plan of cure by the needle. The arm was very much swollen and inflamed, and the constitutional disturbance greatly in excess, all of which admonished me, that any additional irritation would probably end in ulceration of the parts. If then, a cure can be effected of an aneurism, under such untoward circumstances, what might we not expect where the aneurism was slowly progressive—where the system was not much implicated—and where the contiguous parts did not participate in the abnormal action?—*Southern Medical and Surgical Journal, for July.*

*Treatment of Erysipelas, by raw cotton.*—Dr. F. M. Robertson, of Augusta, Geo., reports, in the July number of the Southern Medical and Surgical Journal, two cases of erysipelas, successfully treated by the external application of raw cotton.

He was induced to employ the remedy from the notice of it, by M. Reynaud, of Paris, published in the first volume of the Examiner, and has every reason to be satisfied with its efficacy.

---

*Treatment of Scarlet Fever; contained in a letter to the Editor.* By Edward C. Keckeley, M.D. Charleston, S. C.—Believing it to be the duty of every physician to add all he can to the stock of medical facts, I send the plan of practice which has been pursued by me in scarlet fever. When first called to prescribe for a case, I candidly confess, that I obeyed the call with reluctance. I had been induced to consider the disease as almost an opprobrium medicorum. I determined to attempt a tract which was not altogether beaten smooth. I found it so pleasant, that I now travel it without the slightest fear of not arriving at the full consummation of my fondest hopes—the speedy relief of my patient.

The division of scarlet fever which has been made by Dr. Dewees, answers all practical purposes. The "scarlatina simplex, or simple constitutional disease, without any morbid affection of the throat," requires very little medical treatment. It will run its course, and the patient get well without any thing being done. I have seen several cases terminate in this way, without the least attention being paid to them. This was the case in my own family. When medicine is necessary, the bowels should be gently operated on with small doses of calomel, and Epsom salts, and magnesia. The diet should be antiphlogistic. In the anginose state of the disease, the treatment must be commenced with the full emetic operation of ipecac. The emetic may be repeated daily, or even twice in a day. This is the anchor of our hopes. I have seen the disease put a stop to after one full emetic operation. The bowels should be operated on daily by small doses of calomel: two grains to be given every hour until the object is accomplished. So much for the constitutional treatment. Not the least important part of the treatment is the faithful use of suitable gargles. When ulceration has not taken place, I have derived the greatest advantage from the capsicum. It may be made into a tea with hot water. It, I think, effects more when digested for a time in vinegar. After the ulceration has taken place, no mixture as a gargle is superior to the following: 8 oz. solution of Gum Arab., ʒj. Spts. Turpentine, M. I have used this, instead of the capsicum, with the happiest effects. The medicinal virtues of the turpentine are not known, and will probably long remain so, in consequence of its being an article of home production. Medical men too generally undervalue our vegetable productions. In addition to the preceding, external stimulant applications to the throat should not be forgotten. For this purpose, the throat should be rubbed frequently through the day with the following liniment: to ℥j. Olive Oil, add ʒj. Tinc. Capsicum, M. At night a light woollen bag filled with warm ashes should be applied around the neck; and through the day, a piece of flannel. I have seen much benefit derived from the use of the foot bath at bed time. In the malignant form, the same treatment is to be pursued as in the preceding variety. Should any untoward symptom arise, the physician must apply the proper remedy. For the dropsical affection which sometimes supervenes, I generally use small doses of capsicum, combined with sup. carb. soda. No peculiarity of treatment is necessary. In the management of scarlet fever, the greatest attention must be paid by the physician and nurse. From the trouble attendant upon every case, the nurse requires to be closely watched. A good rule to be strictly observed is, whatever is done, let it be done *quickly* and *faithfully*. I have not the least doubt that nearly all of the cases which terminate fatally, are the result either of improper practice, too long delay, or gross neglect on the part of the nurse. I hold scarlet fever to be one among the most easily managed diseases with which the physician has to contend; but neither of the above three faults must be present. For the above plan I claim unlimited success—out of about twenty cases one died, and this one was the result of neglect by the nurse, until it became too late for any thing to be done. I forgot to mention that cold drinks are to be strictly forbidden. Avoid the external application of water, as you would the sting of a poisonous serpent.—*Ibid.*

